# Potential long-term impact of “On The Move” group-exercise program on falls and healthcare utilization in older adults: an exploratory analysis of a randomized controlled trial

**DOI:** 10.1186/s12877-020-1506-3

**Published:** 2020-03-16

**Authors:** Peter C. Coyle, Subashan Perera, Steven M. Albert, Janet K. Freburger, Jessie M. VanSwearingen, Jennifer S. Brach

**Affiliations:** 1 Department of Physical Therapy, University of Delaware, Newark, DE USA; 2 Department of Physical Therapy, University of Pittsburgh, Pittsburgh, PA USA; 3 Department of Medicine (Division of Geriatric Medicine), University of Pittsburgh, Pittsburgh, PA USA; 4 Department of Behavioral and Community Health Science, University of Pittsburgh, Pittsburgh, PA USA

**Keywords:** Aging, Healthcare utilization, Falls, Prevention, Wellness, Mobility

## Abstract

**Background:**

Wellness program participation may reduce the risk of falling, emergency department-use, and hospitalization among older adults. “On the Move” (OTM), a community-based group exercise program focused on the timing and coordination of walking, improved mobility in older adults, but its impact on falls, emergency department-use, and hospitalizations remains unclear. The aim of this preliminary study was to investigate the potential long-term effects that OTM may have on downstream, tertiary outcomes.

**Methods:**

We conducted a secondary analysis of a cluster-randomized, single-blind intervention trial, which compared two community-based, group exercise programs: OTM and a seated exercise program on strength, endurance, and flexibility (i.e. ‘usual-care’). Program classes met for 50 min/session, 2 sessions/week, for 12 weeks. Older adults (≥65 years), with the ability to ambulate independently at ≥0.60 m/s were recruited. Self-reported incidence of falls, emergency department visitation, and hospitalization were assessed using automated monthly phone calls for the year following intervention completion. Participants with ≥1 completed phone call were included in the analyses. Incidence rate ratios (IRRs) and 95% confidence intervals (CIs) were calculated (reference = usual-care).

**Results:**

Participants (*n* = 248) were similar on baseline characteristics and number of monthly phone calls completed. Participants in the seated exercise program attended an average of 2.9 more classes (*p* = .017). Of note, all results were not statistically significant (i.e. 95% CI overlapped a null value of 1.0). However, point estimates suggest OTM participation resulted in a decreased incidence rate of hospitalization compared to usual-care (IRR = 0.88; 95% CI = 0.59–1.32), and the estimates strengthened when controlling for between-group differences in attendance (adjusted IRR = 0.82; 95% CI = 0.56–1.21). Falls and emergency department visit incidence rates were initially greater for OTM participants, but decreased after controlling for attendance (adjusted IRR = 1.08; 95% CI = 0.72–1.62 and adjusted IRR = 0.96; 95% CI = 0.55–1.66, respectively).

**Conclusion:**

Compared to a community-based seated group exercise program, participation in OTM may result in a reduced risk of hospitalization. When OTM is adhered to, the risk for falling and hospitalizations are attenuated. However, definitive conclusions cannot be made. Nevertheless, it appears that a larger randomized trial, designed to specifically evaluate the impact of OTM on these downstream health outcomes is warranted.

**Trial registration:**

Clinical trials.gov (NCT01986647; prospectively registered on November 18, 2013).

## Background

Aged adults are at a higher risk for chronic disease and falls. Both chronic disease and falls negatively impact quality of life and lead to increased healthcare spending. Older adults account for 50% or more of healthcare spending for ischemic heart disease, hypertension, and falls—three of the five most costly healthcare conditions in the United States [1]. Relative to younger adults, older adults also have the highest rates of emergency department (ED) visits [2] and hospitalizations [3].

Health and wellness programs that promote physical activity are emerging as cost-effective strategies for chronic disease management in older adults [4]. Recently, a large randomized controlled trial established the effectiveness of the On the Move (OTM) community-based exercise program to improve walking. Focused on the timing and coordination of walking [5, 6], OTM improved walking speed and distance in community-dwelling older adults, more than a seated group exercise program commonly available in the community (i.e. usual-care) [5].

Ancillary to that parent trial, participants were followed over the course of a year after intervention completion, to explore the potential impact that the OTM program may have had on downstream health and health services outcomes (i.e. incident self-reported falls, ED visits, and hospitalizations). Because these were secondary outcomes, the parent study was not powered on these outcomes. The overall goal of this study was to explore the potential long-term effects that OTM has on tertiary, health and health services outcomes (i.e. falls, ED visits, and hospitalizations). Randomized controlled trials are resource-intensive; secondary studies, such as this, are helpful in determining if a future trial is warranted. We hypothesized that compared to the usual-care group program, participation in OTM would reduce the risk of these self-reported tertiary, adverse health outcomes over a 12-month follow-up period.

## Methods

### Overview

Details on the original study design, methodology, and primary outcomes are published elsewhere [5, 6]. Briefly, the study was a cluster-randomized, single-blind intervention trial to compare the effectiveness of OTM against a seated group exercise program (i.e. usual-care) for community-dwelling older adults. Both programs were held for 50 min per session, twice weekly, for 12 weeks and were taught by research team staff who were trained exercise professionals, with varying credentials (eg, physical therapists, physical therapy assistants, exercise physiologists). The study was prospectively registered on clinical trials.gov (NCT01986647), on November 18, 2013. All policies and procedures were followed in accordance with the proposal approved by the University of Pittsburgh Institutional Review Board (PRO13090264) and the Helsinki Declaration of the World Medical Association. Signed informed consent was obtained from all participants.

### Participants

Older adults (≥65 years) in the greater Pittsburgh, Pennsylvania area who attended participating senior community centers, or were residing in participating independent living facilities or senior housing, were recruited between April 2014 and January 2016. Participants were excluded if they could not ambulate independently with a gait speed ≥0.60 m/s, were non-English-speaking, were cognitively impaired (i.e. could not follow two-step commands), or were medically unstable [5, 6].

### Randomization

Randomization occurred at the facility-level. Independent living facilities were identified and stratified by several factors (e.g. socioeconomic status, location) to ensure balance during randomization. A statistician who was blinded to outcomes assessment and group assignment, developed and conducted the randomization protocol and process. Senior community centers and senior housing sites were randomized in the order they were recruited, using random block sizes of two and four. Thirty-two sites were included in this study.

### Intervention arms

The exercise classes involved up to 10 participants. The OTM group exercise program included exercises based on motor control principles [7–10], and progressed in difficulty. Exercises used a goal-oriented approach, focusing on stepping and walking patterns to promote timing and coordination, integrated with phases of the gait cycle [9, 11, 12]. Exercise examples included oval, spiral, and serpentine walking patterns, as well as forward, forward-diagonal, and backward stepping. Participants also completed strengthening exercises for the lower extremity muscle groups that are important for walking [9, 11, 12]. Most of the program was conducted in standing.

The usual-care group exercise program was a seated program focused on seated strength, endurance, and flexibility exercises, and it served as an active control arm. Strengthening and endurance exercises focused on upper and lower extremity muscle groups and utilized various techniques to provide resistance (e.g. playground balls, opposite extremity, body weight). Lower extremity strengthening exercises focused on muscles that are important for walking; the upper and lower extremity endurance exercises targeted muscle groups important for physical function, including walking and other activities of daily living.

### Falls, ED visits, & hospitalization measures

After completing the intervention, participants were called monthly for 12 months, via an interactive voice response (IVR) automated system [13], to ascertain whether the participant had fallen, visited the ED, or was admitted to the hospital during the preceding month. Participants that withdrew or were lost to follow-up during the intervention did not receive the monthly follow-up phone calls. The IVR system scheduled calls twice daily (in the late-morning and early-evening) for the 4 days before, and after, the due date. If a participant did not complete a call within the time-window, the data were considered missing, and attempts were made to contact the participant in the next month. The IVR system is ideal for exploratory self-reported outcomes, because of the reduced resources needed to operate it.

The automated message asked if, in the preceding 30 days, participants had fallen (i.e. “landed on the floor or ground and could not stop yourself”); attended the ED for any reason; or, were admitted to the hospital for ≥1 night for any reason. Participants responded “yes” or “no” by pushing buttons on their phone. This method of obtaining regular follow-up data is reliable, valid, and acceptable to older adults [13].

**Fig. 1:**
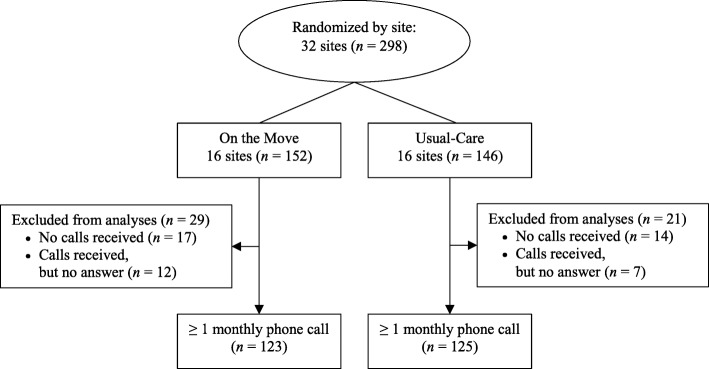
CONSORT diagram for study overview. For flow diagram of the main trial, see reference Brach et al. [5]

The outcomes were the number of months wherein participants experienced a fall, ED visit, and/or hospitalization in the 12 months after the intervention. The number of months that each outcome occurred was counted. Analyses were limited to individuals who completed at least one monthly automated phone call (Fig. 1).

### Baseline and adherence measures

At baseline, participants reported age, sex, race, education level, body mass index (from self-reported height and weight), fear of falling, and history of ≥1 fall over the preceding year. Performance-based mobility assessment included the six-minute walk test [14–16], which is a measure of walking endurance, and gait speed, which was derived from six passes over an instrumented walkway [17–19]. Poorer mobility performance was demonstrated by shorter six-minute walk test distance and slower gait speed. The Duke Comorbidity Index was used to measure comorbidity burden in the following body-systems: cardiovascular, respiratory, musculoskeletal, neurological, general, cancer, diabetes, and visual. Scores on the Duke Comorbidity Index ranged from 0 to 8, where lower scores indicated a lower number of body-systems with comorbidity [20]. Participants also provided global ratings of overall health, balance, and mobility, where ratings ranged from 1 = Excellent to 5 = Poor.

Adherence to the intervention, a surrogate measure of intervention-dose, was monitored using a class roster for attendance at each session. The number of sessions attended was compiled for each participant.

### Statistical analyses

We used appropriate descriptive statistics to summarize data by whether they were included in the analytic subsample (i.e. completed at least one automated monthly phone call) and by intervention group. To account for clustering of participants by facility, we used linear mixed models (LMM) and generalized estimating equations (GEE) models for comparing continuous and categorical variables, respectively. We compared baseline characteristics of those who completed at least one automated monthly phone call vs. those who did not complete any phone calls (ie, included vs. excluded in the analytic subsample); of those who completed at least one phone call (ie, included in the analytic subsample), we compared baseline characteristics by intervention group (OTM vs. usual-care). For continuous variables, we fitted a series of LMMs with each continuous measure as the dependent variable, intervention group assignment as a dichotomous fixed effect, and a facility random effect to account for clustering. To make the same comparisons for dichotomous variables, we fit a series of GEE models with a binomial distribution, logit link function, intervention group assignment as a dichotomous independent variable, and an exchangeable working correlation structure to account for clustering. Negative binomial GEE models, with each outcome (ie, falls, emergency department visits, and hospitalizations) as the dependent variable and intervention group assignment as the independent variable, were fitted to compare the effectiveness of the interventions. Incident rates (i.e. the number of months each outcome occurred per 1000 person-months for each intervention group) and incident rate ratios (IRRs) with 95% confidence intervals (CIs) were calculated. Because class attendance varied by intervention arm, the number of classes attended was added to the model as a covariate to obtain adjusted IRRs. We used SAS version 9.3 (SAS Institute, Inc., Cary, North Carolina) for all statistical analyses, with MIXED and GENMOD procedures for main analyses.

## Results

Two hundred and ninety-eight individuals were randomized to either OTM (*n* = 152) or usual-care (*n* = 146). Compared to older adults with no monthly automated phone calls completed (*n* = 50; 16.8%), those who completed at least one monthly phone call (*n* = 248; 83.2%) were similar on almost all baseline characteristics but had more comorbidities (2.9 vs. 2.4 body system domains; *p* = 0.010).

The proportions of individuals who completed at least one monthly automated phone call for both intervention arms were similar for OTM (*n* = 123, 80.9%) and usual-care (*n* = 125, 85.6%)] (Table 1). Baseline characteristics and mean number of monthly automated calls completed were also similar between intervention arms (mean ± SD = 8.2 ± 3.9 vs. 8.9 ± 3.5 for OTM and usual-care, respectively; *p* = 0.304). Older adults in the OTM arm attended significantly fewer intervention sessions than those in the usual-care arm (mean ± SD = 16.7 ± 7.6 vs. 19.6 ± 5.6, respectively; *p* = 0.017).

**Table 1:** Descriptive characteristics

	OTM (***n*** = 123)	Usual-care (***n*** = 125)	***p***-value
	Mean ± SD or N (%)		
Age (y)	79.4 ± 8.3	81.3 ± 7.6	.725
Sex (female)	108 (87.8)	102 (81.6)	.260
BMI (kg/m^2^)	28.6 ± 6.4	27.2 ± 5.3	.273
Comorbidity Index (0–8)	3.0 ± 1.4	2.8 ± 1.5	.548
Education (some college or more)	52 (42.3)	69 (55.2)	.355
Race (white)	107 (87.0)	104 (83.2)	.652
Global health (very good/excellent)	64 (52.0)	63 (50.4)	.831
Global mobility (very good/excellent)	72 (58.5)	77 (61.6)	.606
Global balance (very good/excellent)	42 (34.2)	43 (34.4)	.984
6MWT (m)	272.8 ± 88.2	286.0 ± 89.9	.291
Gait speed (m/s)	0.92 ± 0.21	0.94 ± 0.21	.422
Fear of falling (yes)	46 (37.4)	50 (40.0)	.591
Fall history (≥1 in prior year)	36 (29.3)	34 (27.2)	.735
Intervention sessions attended (0–24)	16.7 ± 7.6	19.6 ± 5.6	.017
Monthly calls completed (0–12)	8.2 ± 3.9	8.9 ± 3.5	.304

All characteristics are from baseline (pre-intervention) assessment, except for those pertaining to intervention session attendance and monthly phone calls

Abbreviations: *OTM* On the Move, *SD* standard deviation, *BMI* (body mass index), *6MWT* (six-minute walk test)

For OTM, there were 87 fall-months from 42 distinct participants (34.1%), 49 ED visit-months from 30 distinct participants (24.4%), and 44 hospitalization-months from 27 distinct participants (22.0%). For usual-care, there were 77 fall-months from 49 distinct participants (39.2%), 53 ED visit-months from 31 distinct participants (24.8%), and 54 hospitalization-months from 38 distinct participants (30.4%). Incidence rates of hospitalization were slightly lower in the OTM than the usual-care group, whereas incidence rates of falls and ED visits were slightly higher in the OTM group (Table 2). Thus, those in the OTM group had a lower incidence rate of hospitalizations compared to the usual-care group (IRR = .88; 95% CI = 0.59–1.32), which decreased further when adjusting for attendance (adjusted IRR==0.82; 95% CI = 0.56–1.21). Although unadjusted incidence of both falls and emergency department visits were higher in the OTM group, these results were attenuated when controlling for class attendance (adjusted IRR = 1.08; 95%CI = 0.72–1.62 and adjusted IRR = 0.96; 95% CI = 0.55–1.66, respectively). It is important to note that all IRR 95% CI estimates overlapped with a value of 1.0, indicating statistical non-significance.

**Table 2:** Incident rates and rate ratios (ref = usual-care) of falls and healthcare utilization

	OTM		Usual-care			
	Total months outcome occurred	Incidence Rate^**a**^	Total months outcome occurred	Incidence Rate^**a**^	IRR (95% CI)	aIRR (95% CI)
Falls	87	15.0	77	11.4	1.23 (0.80–1.90)	1.08 (0.72–1.62)
ED Visit	49	8.5	53	7.8	1.04 (0.61–1.77)	0.96 (0.55–1.66)
Hospitalization	44	7.6	54	8.0	0.88 (0.59–1.32)	0.82 (0.56–1.21)

Abbreviations: *OTM* On the Move, *IRR* incident rate ratio, *aIRR* incident rate ratio adjusted for attendance, *CI* confidence interval, *ED* emergency department

^a^units = outcome-month per 1000 person-months

## Discussion

Our study is the first to explore the potential impact of a new community-based exercise program for older adults, OTM, on downstream health and healthcare utilization outcomes. Of note, older adults who participated in the OTM intervention had a 12% reduction in the risk of hospitalization within the 12 months following the intervention, compared to those who received a usual-care seated group exercise program. Rates of hospitalizations for older adults were 265/1000 in 2015 [3], with an average estimated cost of $12,300 in 2010 [21]. Based on these findings, participation in OTM may potentially impact this costly and prevalent outcome (i.e. hospitalizations in older adults). This study, however, was not originally designed to investigate these hypotheses, and the incidence of these outcomes was low; thus, it is not surprising that our results lacked statistical significance. A much larger sample size would be needed to obtain appropriate statistical power. It is also possible that the incidence between groups is, in reality, similar, and OTM does not lessen the risk of these outcomes compared to Usual-Care. Regardless, the trends found in these analyses warrant further investigation to fully explore this research question, and to make definitive conclusions.

A significant difference in the number of classes attended existed between the OTM (16.7 ± 7.6) and usual-care (19.6 ± 5.6) groups (*p* = .017). To explore the per-protocol impact of OTM vs. usual-care, we also conducted analyses adjusted for class attendance. While the unadjusted analyses yielded estimates that indicated OTM participation was associated with an *increased* risk of falling and ED visits, the adjusted analyses suggested a trend that attenuated this effect, such that OTM participation was associated with a slightly reduced risk of ED visitation. Of note, though the risk of falling was attenuated in the adjusted analyses, the results still indicated that OTM participation was associated with a slightly greater risk of falling.

Future studies should focus on improving the adherence to OTM, while also attaining appropriate power.

The specific mechanisms by which OTM may lead to a reduction in hospitalization also needs further exploration. The original intent of OTM was to improve mobility-related outcomes (i.e. walking speed and distance), which it was successful in accomplishing [5]. Mobility limitations are associated with increased healthcare utilization [22, 23], and reductions in mobility limitations may lead to greater physical activity and reduced sedentary behavior. Cardiac (e.g. heart failure) and pulmonary (e.g. chronic obstructive pulmonary disease) are leading causes for hospitalization following ED visit among older adults [2], and may be worsened by physical inactivity. It’s possible that more physical activity and less sitting, which may be facilitated through improved mobility, may lead to a slowing of the disease process and/or a reduction in episodes of exacerbation for these conditions. Also, participation in OTM may have led to reduced hospitalization through other, albeit related, pathways. For instance, individuals in OTM spent a larger proportion of class-time on their feet and walking. It is possible that OTM participants acquired better bone health, greater confidence in walking and being more physically active, and a better insight into managing their own mobility, regardless of its impact on performance-based mobility. It is conceivable that any of these mechanisms could result in a reduction in hospitalizations. These analyses do not allow us to establish a causal pathway, and an accurate mediation analysis is not possible given the exploratory nature of these data.

Over a year of follow-up, OTM participants (mean age = 79.4 years) had 8.4% less participants hospitalized, compared to individuals who participated in usual-care (OTM = 22.0% vs. usual-care = 30.4%). Over a two-year follow-up, Nguyen et al. found that providing older adults (mean age = 73.0 years) with access to local fitness centers through an insurance benefit, resulted in 0.1–2.3% less hospitalizations for individuals who participated compared to individuals who did not participate [24]. Ackermann et al. found that participation in another structured, community-based exercise program, which focused on strength, aerobic, flexibility, and balance training, resulted in 0.2–0.4% less hospitalizations, among community-dwelling older adults (mean age = 75.7 years) who participated compared to older adults who did not participate [25]. Further, it should be noted that our study used an active control group (ie, usual-care group exercise), whereas a passive control group was used in the studies by Ackermann and Nguyen. We would expect smaller effect sizes when comparing two active groups (ie, exercise programs), as opposed to comparing individuals who did or did not participate in an intervention, but the effect on hospitalizations was larger in this trial. However, caution should be exercised in truly comparing studies. Our study was a randomized controlled trial, with intervention arms that were led by experienced instructors, whereas the studies by Ackermann and Nguyen were prospective cohort studies, investigating the impact of these programs after widespread implementation. Compared to more ideal research environments, real-world effect sizes tend to be attenuated.

### Study limitations

Other limitations relative to our study should be noted. First and foremost, these estimates should be interpreted with caution, considering their lack of statistical significance (i.e. 95% CIs that overlapped with a null value of 1.0) and precision (i.e. large 95% CIs). The trial was originally designed to detect differences in mobility-related outcomes and, thus, lacked the statistical power to make definitive conclusions. Regardless, rather than drawing definitive conclusions, the aim of the current study was to simply explore trends that may warrant further investigation in a future trial that is appropriately powered for these analyses.

Second, only the findings relative to hospitalizations favored OTM in the unadjusted analyses, but this may have been due to lower adherence to the OTM intervention. Because this was an exploratory study, however, we elected to perform separate analyses controlling for class attendance (i.e. intervention-dose); this approach allowed us to examine the impact of the intervention when completed as prescribed (i.e. per-protocol). The per-protocol adjusted analyses saw decreasing trends in IRRs for falls and ED visits; in the case of ED Visits, the IRR went from 1.04 to 0.96, favoring OTM participation. These results suggest that OTM, when completed as prescribed, may reduce the risk of ED visitation, in addition to hospitalization. However, even after adjustment, the results were still not statistically significant, and those who participated in OTM were still at a slightly greater risk of falling (i.e. IRR decreased from 1.23 to 1.08). Barriers to OTM class attendance should be explored in more depth and, if possible, intervened upon to optimize the benefits of the program.

Third, there was a degree of missing data in the analytic subsample; on average, approximately 8 calls (of a maximum of 12) were completed for each intervention group. However, the rate of call response was similar between arms; thus, we feel confident in the comparisons we conducted. In future studies, an approach to supplement the IVR system (i.e. option for manual calling by research staff) may be needed to minimize missing data.

## Conclusion

Compared to a usual-care community-based group exercise program, participation in OTM may result in a reduced risk of hospitalization in the year following intervention completion. In addition, adjusted analyses indicate that when OTM is adhered to as prescribed, the risk for all tertiary outcomes decreases further. However, given the limitations and exploratory nature of our study, which may have contributed to our statistically non-significant findings, definitive conclusions cannot be made; thus, further investigation is needed. Given these preliminary findings, it appears that a larger randomized trial, designed to specifically evaluate OTM’s effectiveness in reducing the risk of these downstream healthcare outcomes is warranted.

## Data Availability

For data and materials, please contact the corresponding author on reasonable request.

## Abbreviations

CI: confidence interval
ED: emergency department
GEE: generalized estimating equations
IRR: incident rate ratio
LMM: linear mixed models
OTM: On the Move
